# Outcome of revision hip arthroplasty in patients younger than 55 years: an analysis of 1,037 revisions in the Dutch Arthroplasty Register

**DOI:** 10.1080/17453674.2019.1708655

**Published:** 2020-01-13

**Authors:** Martijn F L Kuijpers, Gerjon Hannink, Liza N van Steenbergen, B Willem Schreurs

**Affiliations:** a Radboud University Medical Center, Radboud Institute for Health Sciences, Department of Orthopaedics, Nijmegen;; b Radboud University Medical Center, Radboud Institute for Health Sciences, Department of Operating Rooms, Nijmegen;; c Dutch Arthroplasty Register (Landelijke Registratie Orthopedische Implantaten), ‘s Hertogenbosch, the Netherlands

## Abstract

Background and purpose — The increasing use of hip arthroplasties in young patients will inevitably lead to more revision procedures at younger ages, especially as the outcome of their primary procedures is inferior compared with older patients. However, data on the outcome of revision hip arthroplasty in young patients are limited. We determined the failure rates of revised hip prostheses performed in patients under 55 years using Dutch Arthroplasty Register (LROI) data.

Patients and methods — All 1,037 revised hip arthroplasty procedures in patients under 55 years at the moment of revision registered in the LROI during the years 2007–2018 were included. Kaplan–Meier survival analyses were used to calculate failure rates of revised hip arthroplasties with endpoint re-revision for any reason. Competing risk analyses were used to determine the probability of re-revision for the endpoints infection, dislocation, acetabular and femoral loosening, while other reasons for revisions and death were considered as competing risks.

Results — Mean follow-up of revision procedures was 3.9 years (0.1–12). 214 re-revisions were registered. The most common reason for the index revision was dislocation (20%); the most common reason for re-revision was infection (35%). The 5-year failure rate of revised hip prostheses was 22% (95% CI 19–25), and the 10-year failure rate was 28% (CI 24–33). The 10-year cumulative failure rates of index revisions with endpoint re-revision for infection was 7.8% (CI 6.1–9.7), acetabular loosening 7.0% (CI 4.1–11), dislocation 3.8% (CI 2.6–5.2), and femoral loosening 2.7% (CI 1.6–4.1). The 10-year implant failure rate of index revisions for infection was 45% (CI 37–55) with endpoint re-revision for any reason.

Interpretation — Failure rate of revised hip prostheses in patients under 55 years is worrisome, especially regarding index revisions due to infection. This information facilitates realistic expectations for these young patients at the time of primary THA.

Total hip arthroplasty (THA) is used more and more in younger patients (Kurtz et al. 2009, Otten et al. 2010). Projections show that by the year 2030, more than half of all primary THA will be placed in patients younger than 65 years of age, with the biggest increase expected in patients between 45 and 54 years old (Kurtz et al. 2009).

However, the outcome of primary THA in young patients is inferior compared with older patients (Walker et al. 2016, AOANJJR 2018, NJR 2018). Due to this increase in number of primary THA in young patients, and the inferior outcome, an increase in the number of revision arthroplasties is inevitable in young patients. Bayliss et al. (2017) have already shown that the lifetime risk of revision (LTRR) after THA increases with decreasing age at the time of primary surgery, with LTRR reaching almost 30% in patients between 50 and 54 years of age.

Data on survivorship of revision procedures in young patients are limited. There are a few studies available that assessed the survival of revision procedures. The outcome of these studies was disappointing, with reported survival rates between 36% and 87% at 10-year follow-up (Girard et al. 2011, Adelani et al. 2014, Lee et al. 2014, Te Stroet et al. 2015, Beckmann et al. 2018). Besides this inferior outcome, most of these studies were single-center studies and had small sample sizes. In addition, previous reports focused primarily on implant design (Beckmann et al. 2018) or surgical technique (Comba et al. 2009), and there is a lack of reports focusing on the outcome of revisions in young patients using registry data. Understanding of the extent of the problem in revision arthroplasty in young patients is important, not only to reduce the number of re-revisions, but also to provide realistic expectations for this young patient group (Schreurs and Hannink 2017).

Therefore, we determined the failure rate of revision hip arthroplasty performed in patients younger than 55 years of age using data from the Dutch Arthroplasty Register (LROI).  

## Patients and methods

The LROI (Dutch Arthroplasty Register) is a nationwide population-based register collecting data on arthroplasties. Initiated by the Dutch Orthopaedic Association, data collection started in 2007. The database has coverage of all Dutch hospitals, a completeness of over 95% of primary THA and 88% for revision arthroplasty (van Steenbergen et al. 2015), and 98% for both primary and revision THA in recent years (LROI 2018). Prosthesis characteristics are derived from an implant library within the LROI, which contains core characteristics of prostheses used in the Netherlands based on the article number (van Steenbergen et al. 2015).

**Figure 1.**
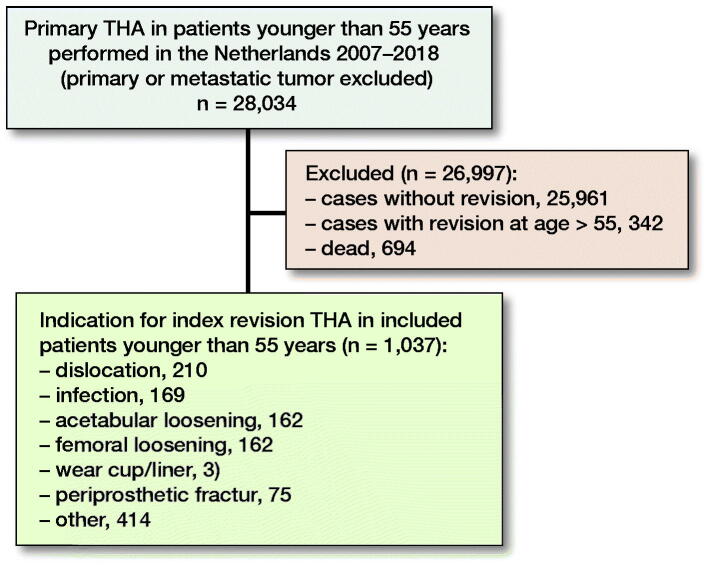
Flowchart of patient selection.

For this study, we selected all primary THA placed between January 1, 2007 and December 31, 2018 in patients younger than 55 years in the Netherlands (n = 28,034). Primary THAs performed because of a tumor (primary or metastatic) were excluded. Next, we included the subsequent revision procedures from this cohort in patients who were younger than 55 at time of their index revision procedure (n = 1,037) (Figure 1). A revision procedure was defined as an exchange of at least 1 of the components of the implant. Within the LROI, 3 revision categories are distinguished: (1) total revision—indicates a revision of the complete prosthesis, replacing both the acetabular and femoral components, (2) major partial revision—indicates a revision procedure where at least the femoral or the acetabular component is revised, and (3) minor partial revision—indicates a revision procedure where only the head and/or the liner of the prosthesis is replaced. 2-stage revisions and Girdlestone procedures are registered at time of the definitive re-implantation of the prosthesis/components. This study was conducted and reported according to STROBE guidelines. 

### Statistics

Survival time of the implant inserted during the revision procedure was calculated as time from the index revision procedure to re-revision, death of the patient or the end of study follow-up (January 1, 2019). In case of a Girdlestone procedure during the revision procedure, survival time is calculated between the re-implantation of the prosthesis (index revision) and re-revision, death of the patient, or the end of study follow-up. Kaplan–Meier survival analyses were used to estimate the survival of the implants inserted during all index revision procedures with endpoint re-revision for any reason. Results of Kaplan–Meier analyses were reported as cumulative failure rate (1 – KM) with 95% confidence intervals (CI).

Next, implant survival with endpoint re-revision for any reason for the following subgroups: (1) revision category (i.e., total revision, major, and minor partial revision), and (2) reason for index revision (i.e., acetabular loosening, dislocation, and infection) were estimated using Kaplan–Meier survival analyses. Log-rank tests were used to test for differences in survival between groups.

Using competing risk analyses, the probabilities of re-revision with endpoint re-revision for acetabular and femoral loosening, dislocation, and infection were determined, where death and other reasons for re-revision were considered as competing events. All analyses were performed using R version 3.5.1 (R Foundation for Statistical Computing, Vienna, Austria).

### Ethics, funding, data sharing, and potential conflict of interest

Ethical approval was not applicable, as all data were received completely anonymous. This study was funded by the Van Rens Foundation, the Netherlands (VRF2017-009). The funding body had no role in the design of the study, data collection, analysis and interpretation, or in writing of the manuscript. Data are available from the LROI (Dutch Arthroplasty Registry) but restrictions apply to the availability of these data, which were used under license for the current study. The authors declare that they have no competing interests. 

## Results

### Characteristics of the study population

Between January 1, 2007 and December 31, 2018, 1,037 index revisions (number of patients = 1,019) were registered in the LROI. Median age at time of revision was 49 years (18–54), and 53% were females. Other patient and implant characteristics are given in Table 1.

**Table 1.** Patient characteristics of 1,037 revisions including percentages in parantheses

Factor	Index revisions (n = 1,037)
Age (years)^a^	49 (18–54)
Sex	
Female	548 (53)
Male	488 (47)
Missing	1 (0.1)
ASA classification	
I	401 (39)
II	470 (45)
III–IV	115 (11)
Missing	51 (5)
Reason for index revision^b^	
Loosening acetabulum	162 (16)
Loosening femur	162 (16)
Dislocation	210 (20)
Infection	169 (16)
Wear cup/liner	35 (4)
Periprosthetic fracture	75 (7)
Other^c^	414 (40)

a Median (range)

b Total is more than 100%, as patients can have more than 1 reason for revision.

c Includes periarticular ossification, symptomatic MoM, and Girdlestone procedures.

The most common reason for the index revision was dislocation (20%), followed by infection (16%), acetabular loosening (16%), and femoral loosening (16%) (Table 2). The mean follow-up of the index revision procedures was 3.9 years (0.1–11.8).

**Table 2.** Patient characteristics of 214 re-revisions including percentages in paranthesis

Factor	Re-revisions (n = 214)
Age (years) **^a^**	50 (19–58)
Sex	
Female	103 (48)
Male	111 (52)
ASA classification	
I	51 (24)
II	117 (55)
III–IV	36 (17)
Missing	10 (5)
Reason for re-revision**^b^**	
Loosening acetabulum	34 (16)
Loosening femur	21 (10)
Dislocation	34 (16)
Infection	74 (35)
Wear cup/liner	8 (4)
Periprosthetic fracture	7 (3)
Other**^c^**	96 (45)

a–c See Table 1.

Of 1,037 index revisions, 21% of cases had replacement of both the acetabular and femoral component (total revision). In 53% of all index revisions, there was at least a replacement of the acetabular or femoral component (major partial revision), where 18% were a revision of the head and/or a replacement of the liner (minor partial revision). Of all major partial revisions, 57% involved a cup revision, where in 43% the femoral component was revised. In 8% of all index revisions, the revision category involved either a Girdlestone procedure, was reported as other, or was missing. In 3 cases, there was no re-implantation of a prosthesis after a Girdlestone procedure. Therefore, these cases were excluded from the survival analyses.

There were 169 index revisions because of an infection. Of these, 44% cases had a minor partial revision (replacing only the head or liner), indicating a DAIR procedure. Furthermore, 29% of these cases were registered as a Girdlestone procedure, indicating a 2-stage revision procedure. Additionally, 22% of these cases were registered as total revision, of which 13 cases had Girdlestone as reason for revision. Therefore, these procedures can also be considered as a 2-stage revision, resulting in a total of 37% 2-stage revision procedures. The remaining 14% of total revision procedures were 1-stage revision procedures. There were 4% major partial revisions with reason given as infection; 2 cup revisions and 4 stem revisions, which were also considered as 1-stage revision procedures.

### Re-revision procedures

214 re-revision procedures were registered. The most common reason for re-revision was infection (35%), followed by acetabular loosening (16%) and dislocation (16%) (Table 2).

Of 214 re-revision procedures, 29% had replacement of both the acetabular and femoral component (total re-revision). In 41% cases of all re-revisions, there was at least replacement of the acetabular or femoral component (major partial re-revision), where 18% of cases were revision of the head and/or replacement of the liner (minor partial re-revision). From all major partial re-revisions, 76% involved the cup, whereas 24% involved the femoral component. In 12% of the re-revised hips, the type of re-revision involved a Girdlestone procedure, was reported as other, or was missing. 

### Failure rates of index revisions

Using Kaplan–Meier, the 5-year implant failure rate of the 1,037 index revisions with endpoint re-revision for any reason was 22%. At 10-year follow-up, the implant failure rate was 28%. The 5- and 10-year cumulative failure rates of index revisions with endpoint re-revision for infection were 7.5% and 7.8%. For acetabular loosening, the 5- and 10-year cumulative failure rates were 3.1% and 7.0%. For dislocation, this was 3.8% and 3.8%. For femoral loosening, the 5- and 10-year cumulative failure rates were 2.3% and 2.7% (Table 3).

**Table 3.** Failure rate (%) of all index revisions, by category of revision and by reason for index revision

Factor	5-year failure rate (95% CI)	10-year failure rate (95% CI)
All index revisions with endpoint re-revision for		
any reason	22 (19–25)	28 (24–33)
dislocation	3.8 (2.6–5.2)	3.8 (2.6–5.2)
infection	7.5 (5.9–9.3)	7.8 (6.1–9.6)
acetabular loosening	3.1 (2.1–4.4)	7.0 (4.1–11)
femoral loosening	2.3 (1.5–3.5)	2.7 (1.6–4.1)
Revisions category		
total revision	15 (11–21)	18 (13–21)
major partial revision	16 (13–20)	22 (17–27)
minor partial revision	31 (24–39)	50 (32–73)
Reason for index revision		
infection	45 (37–55)	45 (37–55)
dislocation	22 (16–29)	29 (20–41)
acetabular loosening	22 (16–33)	31 (21–44)
femoral loosening	18 (13–26)	22 (14–34)

### Failure rates by revision category

**Figure 2.**
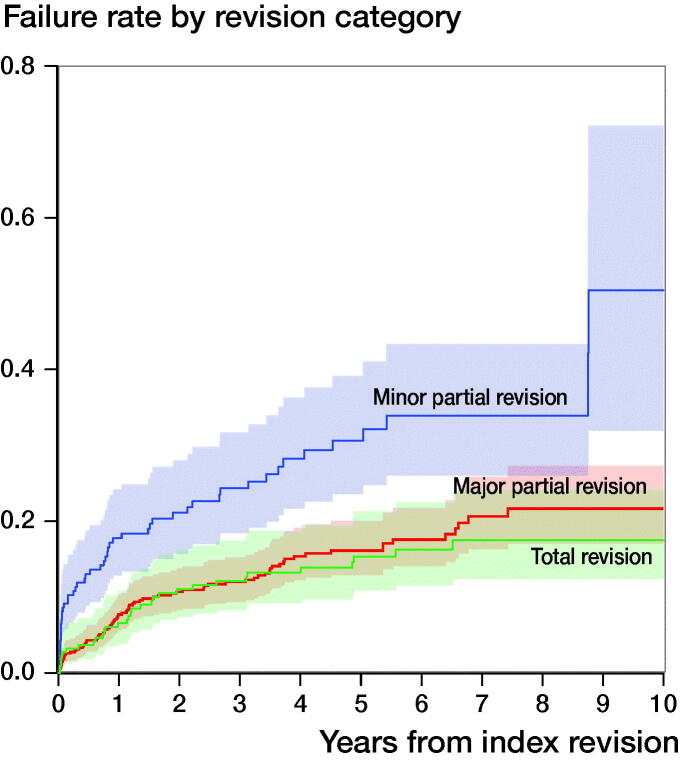
Failure rate by revision category with endpoint re-revision for any reason.

The 5- and 10-year failure rate for total revision procedures was 15% and 18% (CI 12.5–21.2). For major partial revisions, the 5- and 10-year failure rate was 16% and 22%. For minor partial revisions, this was 31% and 50% (Table 3). A log-rank test showed a significant difference in failure between categories of revision (p < 0.001, Figure 2).

### Failure rates by reason for index revision

**Figure 3.**
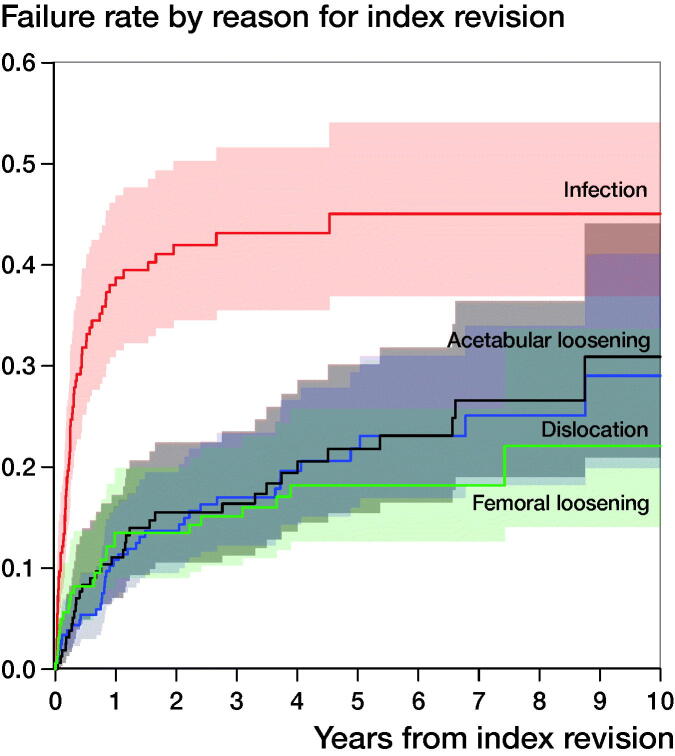
Failure rate by reason for index revision with endpoint re-revision for any reason.

The failure rate of index revisions with reason given as infection was high. Using Kaplan–Meier analyses, the 5-year implant failure rate of these procedures, with endpoint re-revision for any reason, was 45%. At 10 years, the failure rate was 45%. For index revisions with reason given as dislocation, the 5-year failure rate with endpoint re-revision for any reason was 22%, and the failure rate at 10 years was 29%. For index revisions with reason given as acetabular loosening, the 5- and 10-year implant failure rate was 22% and 31%. For index revisions with reason given as femoral loosening, the 5-year failure rate with endpoint re-revision for any reason was 18%, and the failure rate at 10 years was 22% (Table 3, Figure 3).

Additionally, patients who had an index revision with reason given as infection had a high cumulative failure rate for endpoint re-revision for a recurrent infection. At 5 years, more than 30% of all patients underwent a re-revision procedure with reason given as infection (cumulative failure rate 30%; CI 23–38). The competing risk analysis showed that the cumulative failure for a re-revision with recurrent reason was much lower for patients who underwent an index revision with reason given as dislocation, acetabular loosening, or femoral loosening. Only 8% of patients who underwent their index revision procedure with reason given as dislocation had a re-revision for another dislocation (cumulative failure rate 8.3%, CI 4.7–13). For acetabular loosening, this was only 5.3% (CI 2.3–10) at 5 years. For femoral loosening, the cumulative failure rate at 5 years for recurrent loosening of the femur was 5.0% (CI 2.2–9.5). 

## Discussion

Our analysis showed a 5-year failure rate of index revision procedures with endpoint re-revision for any reason of 22% (CI 19–25), and 28% (CI 24–33) at 10-year follow-up.

### Comparison with literature

Survival at 5-year follow-up was lower when compared with the available literature on young patients (Lee et al. 2014, Gromov et al. 2015, Te Stroet et al. 2015). Few papers analyzed the mid- to long-term survival of revisions in this patient group, the number of included patients in these studies was limited, and focus was primarily on implant design.

Te Stroet et al. (2015) reported a survival rate of 87% after follow-up of 10 years in a single-center study with only 34 revision procedures. Lee et al. (2014) reported a survival rate of 63% at 10-year follow-up, whereas survival at 5 years was approximately 88%. Several studies assessed the survival of revision hip arthroplasty in older patients, where reported survival varied between 81% and 83% at 5 years (Jafari et al. 2010, Ong et al. 2010) and 72% at 10 years (Lie et al. 2004). However, these results are relatively dated, and reason for revision was not reported in all studies, which makes comparison difficult.

The most prevalent reason for the index revision was dislocation, whereas the most common reason for re-revision was infection. The rate of infections in the index revision procedures was 16%, which increased to 35% in all re-revision procedures. That infections are more prevalent as reason for re-revisions when compared with index revision procedures shows that management of infections plays an important role for prevention of re-revisions. Additionally, it is known that dislocation is a common complication associated with THA (Gwam et al. 2017, Seagrave et al. 2017, Rajaee et al. 2018). This was confirmed in our data, where dislocation was the most frequent reason for index revisions (16%). However, in re-revision procedures, dislocation as reason for re-revision is less pronounced when compared with infections. For prevention of re-revisions, the focus should be on treatment of infections (Berry 2017).

Moreover, the survival of index revisions with reason given as infection was poor. At 5 years, almost half of all revised hips due to an infection resulted in re-revision. Furthermore, the number of re-infections was high in this group. Within 5 years, approximately 30% of all index revisions with reason given as infection underwent re-revision for a re-infection. For other reasons for revision, these numbers were much lower, with only 8% for dislocation, and 5% for both acetabular and femoral loosening.

We found a substantial difference in failure rates between the different categories of revision. Approximately 70% of all index revisions were a partial revision, where in the majority of these procedures either the cup or the stem was replaced (major partial revision). Failure rate of the minor partial revisions (replacement of head and/or insert) was higher when compared with major partial revisions or total revisions, which is supported by data from the Swedish Hip Arthroplasty Register (Mohaddes et al. 2013). A possible explanation for this might be the high percentage of index revisions with reason given as infection using this method, or the exchange of heads to prevent further dislocations. Of all minor partial revisions, 40% were DAIR procedures (minor partial revisions for infection). The numbers of index revisions with reason given as infection using a total revision or a major partial revision were much lower, at respectively 17% and 1%. Nevertheless, the effectiveness of the minor partial revision should be reconsidered, as survival of this revision category is lower. 

### Limitations and strengths

The completeness of revision hip arthroplasty in the Dutch Arthroplasty Register is lower compared with the completeness of primary THA, especially in the period 2007–2009, when there was no complete coverage of all Dutch hospitals. Second, there is most likely an under-registration of infections in the registry, as reoperations for infection without replacement of any of the components are not registered in the LROI (Lindgren et al. 2014, Gundtoft et al. 2015, SHAR 2015). In addition, since the outcome of index revisions due to infection is poor, information related to use of antibiotics (e.g., type of antibiotics, use of antibiotic-loaded bone cement, and adherence to guidelines on antibiotics administration) would be particularly valuable to obtain insight into this serious problem. Unfortunately, this information is not available from the Dutch Arthroplasty Register.

A strength is that, compared with literature, our analysis includes a much larger number of revision procedures.

## Conclusion

The cumulative failure rate in revision hip arthroplasty performed in patients under 55 years is worrisome. In particular, the outcome of index revisions due to infection is alarming, with a failure rate of 45% at 10-year follow-up. Moreover, within 5 years, 30% of all patients with an index revision for infection underwent a re-revision procedure with reason given as infection. Therefore, in the prevention of (re-)revisions, management of infections should play an essential role.

MK, GH, LN, BS: concept and design. MK, GH, LN, BS: data analysis and interpretation. MK, GH, BS: manuscript preparation. MK, GH, LN, BS: manuscript editing. MK, GH, LN, BS: manuscript review. MK, GH, LN, BS: final approval of the version submitted.

*Acta* thanks Keijo Mäkelä and Maziar Mohaddes for help with peer review of this study.
